# Genomic analysis reveals high virulence and antibiotic resistance amongst phage susceptible *Acinetobacter baumannii*

**DOI:** 10.1038/s41598-020-73123-y

**Published:** 2020-09-30

**Authors:** Udomluk Leungtongkam, Rapee Thummeepak, Thawatchai Kitti, Kannipa Tasanapak, Jintana Wongwigkarn, Kathryn M. Styles, Elizabeth M. H. Wellington, Andrew D. Millard, Antonia P. Sagona, Sutthirat Sitthisak

**Affiliations:** 1 Department of Microbiology and Parasitology, Faculty of Medical Science, Naresuan University, Phitsanulok, 65000 Thailand; 2 Faculty of Oriental Medicine, Chiang Rai College, Chiang Rai, 57000 Thailand; 3 School of Life Sciences, University of Warwick, Coventry, CV4 7AL UK; 4 Department of Genetics and Genome Biology, University of Leicester, Leicester, LE1 7RH UK

**Keywords:** Genetics, Microbiology

## Abstract

In this study, we examined the association between antimicrobial resistance, CRISPR/Cas systems and virulence with phage susceptibility in *Acinetobacter baumannii* and investigated draft genomes of phage susceptible multidrug resistant *A. baumannii* strains from Thailand. We investigated 230 *A. baumannii* strains using 17 lytic *A. baumannii* phages and the phage susceptibility was 46.5% (107/230). Phage susceptibility was also associated with resistance to numerous antibiotics (*p*-value < 0.05). We also found association between biofilm formation and the presence of *ompA* gene among phage susceptible *A. baumannii* strains (*p*-value < 0.05). *A. baumannii* isolates carrying *cas5* or combinations of two or three other *cas* genes, showed a significant increase in phage resistance. Whole-genome sequences of seven phage susceptible *A. baumannii* isolates revealed that six groups of antibiotic resistance genes were carried by all seven phage susceptible *A. baumannii*. All strains carried biofilm associated genes and two strains harbored complete prophages, acquired copper tolerance genes, and CRISPR-associated (*cas*) genes. In conclusion, our data exhibits an association between virulence determinants and biofilm formation among phage susceptible *A. baumannii* strains. These data help to understand the bacterial co-evolution with phages.

## Introduction

*Acinetobacter baumannii* is a major cause of opportunistic infection, especially among immunocompromised patients. The emergence of multidrug-resistant *A. baumannii* (MDR-AB) and even the extensively drug-resistant *A. baumannii* (XDR-AB) has been increasing worldwide, and especially in Thailand and Nepal^1–3^. Thus, alternative treatments against *A. baumannii* infection is urgently needed. Bacteriophages (phages) are good candidates, specifically killing host bacteria resulting in minimal impact on bacterial normal flora and with no known critical side effects^4^. To use phages for therapy, it is important to identify broad host range phages that kill the highest possible number of strains of bacterial species. In addition, it is crucial to understand the host-phage susceptibility mechanism. One of the important mechanisms impacting host range specificity is phage adsorption^5^. This is a crucial step in the infection process, which represents the initial contact between virus and its host and requires phage receptors; outer membrane proteins (OMPs), lipopolysaccharides and teichoic acids^5^. Several phages use the outer membrane protein OmpA as a receptor to infect Gram negative bacteria^6,7^. Alterations to this molecule result in a decrease of bacterial virulence in phage resistance strains^8^. Previous studies reported a positive correlation of antibiotic resistance in *A. baumannii* with phage susceptibility^9,10^. Phage susceptibility represents an evolutionary trade-off in *A. baumannii* strains that were selected for antibiotic resistance, particularly in hospital environments with high antibiotic use^10^. However, the mechanism and genetic basis of phage susceptibility in *A. baumannii* is not completely understood. In this study, we aimed to determine the association between antimicrobial resistance and virulence with phage susceptibility in a large collection of *A. baumannii* strains and to investigate draft genomes of phage susceptible of MDR-AB strains from Thailand to identify antibiotic resistance genes and virulence genes.

## Results

### Characterization and antimicrobial susceptibility profiles

Among 230 *A. baumannii* isolates, the resistance to various antibiotics was as follows; amikacin (53.47%), cefotaxime (80.43%), ceftazidime (83.04%), ceftriaxone (83.91%), cefepime (73.91%), ciprofloxacin (85.22%), gentamicin (63.48%), imipenem (82.17%), meropenem (81.74%), trimethoprim/sulfamethoxazole (65.65%), tetracycline (61.74%), cefoperazone/sulbactam (28.70%), piperacillin/tazobactam (82.60%). Among all *A. baumannii* isolates, 86.52% (199/230) were MDR-AB, 83.49% (192/230) were carbapenem resistant *A. baumannii* (CR-AB) and 12.17% (28/230) were XDR-AB. A total of 28 (12.17%) isolates were non MDR-AB. All isolates were sensitive to colistin and tigecycline.

### Phage susceptibility among MDR-AB, CR-AB, XDR-AB and non MDR-AB

**Figure 1:**
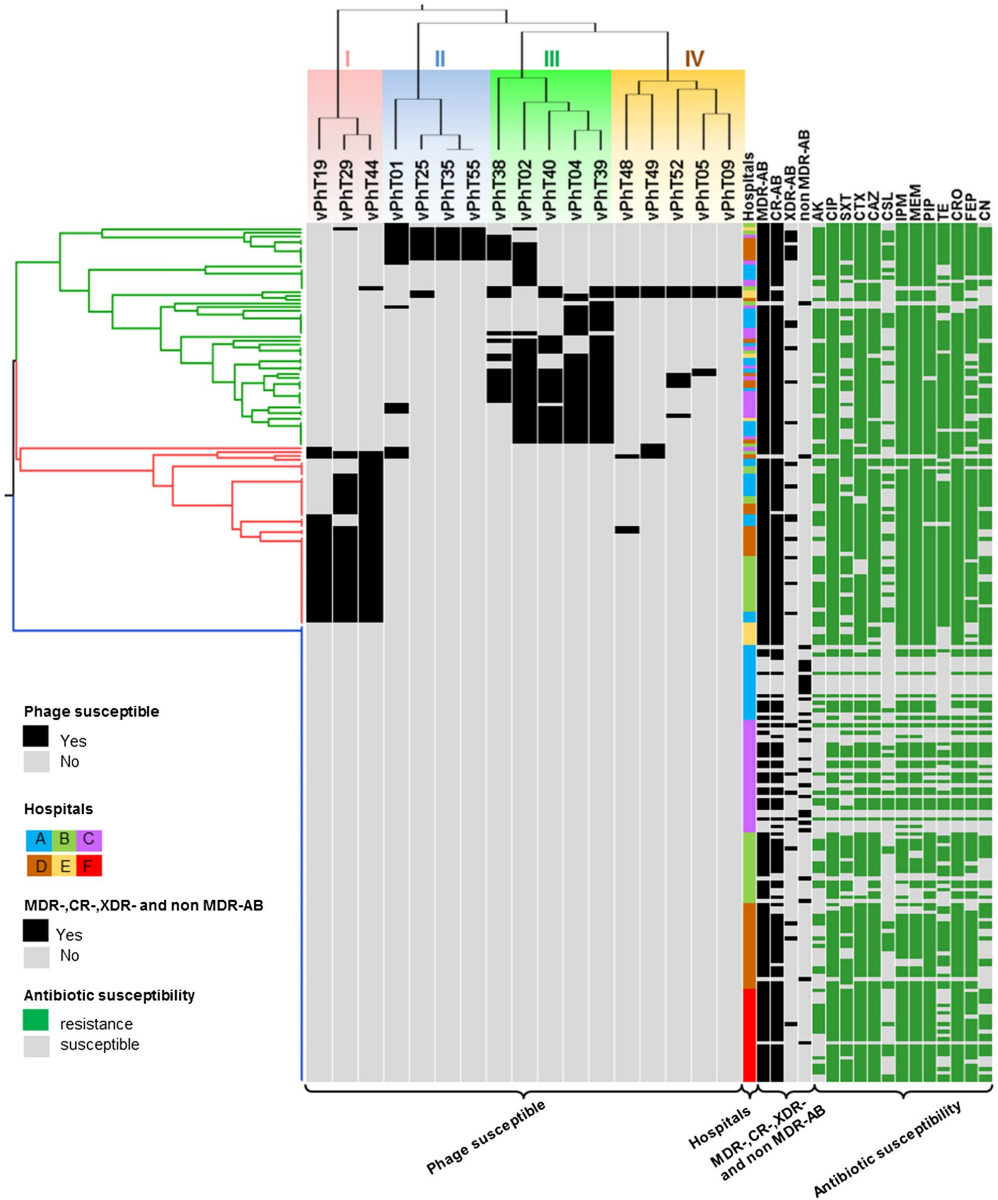
UPGMA dendrogram based on phage susceptibility patterns among 230 *A. baumannii* clinical isolates.) Tree branches represent three bacterial groups of phage susceptibility (Group1: green, Group2 red, Group 3 blue), phage susceptible is displayed as yes (black) and no (gray). Hospitals A-E located in Central, Lower Northern, Northern, East and Northern regions of Thailand, respectively. Hospital F, located in Nepal. Classifications of MDR-, CR-, XDR- and non MDR-AB were presented as yes (black) and no (gray). Antibiotic sensitivity is presented as resistant (green) and susceptible (gray). Antibiotics are abbreviated as follows: *AK* amikacin, *CIP* ciprofloxacin, *SXT* trimethoprim/sulfamethoxazole, *CTX* cefotaxime, *CAZ* ceftazidime, *CSL* cefoperazone/sulbactam, *IPM* imipenem, *MEM* meropenem, *PIP* piperacillin/tazobactam, *TE* tetracycline, *CRO* ceftriaxone, *FEP* cefepime, *CN* gentamicin.

We grouped the 17 phages (Table 1) into four clusters (I, II, III, and IV), depending on their ability to infect 230 *A. baumannii* isolates (Fig. 1). The vPhT19, vPhT29 and vPhT44 phages belonged to cluster I and infected between 13.91 and 20.43% of bacterial hosts. The phages vPhT01, vPhT25, vPhT35 and vPhT55 were grouped into cluster II, showing between 3.91 and 7.83% infectivity. Phages vPhT02, vPhT04, and vPhT39 showed infectivity against 14.78–18.26% of *A. baumannii* isolates and belonged to cluster III. Cluster IV contained five phages (vPhT05, vPhT09, vPhT48, vPhT49 and vPhT52), infecting approximately 1.30–3.48% of *A. baumannii*. Overall phage susceptibility of the 230 *A. baumannii* strains was 46.5% (107/230). The bacteria were divided into three groups. Group 1 (Fig. 1; green) were the bacteria susceptible to a variety of phages and were mostly infected by phages in cluster II, III and IV. In Group 2 (Fig. 1; red) were the bacteria mostly susceptible to phages in cluster I. In Group 3 (Fig. 1; blue) were the phage resistant bacteria. Among the 199 MDR-AB isolates, 105 (52.76%) were infected specifically by at least one phage. Most of the CR-AB (53.64%) and XDR-AB (71.43%) were infected specifically by at least one phage. Only two of twenty-eight of non MDR-AB (7.14%) were phage susceptible strains (Fig. 1). Of the 230 *A. baumannii* strains, 205 were isolated from Thailand and 25 were from Nepal. We found 52.2% (107/205) of *A. baumannii* isolates from Thailand were phage susceptible (Supplementary Table S1). Interestingly, we observed that all 17 phages isolated from Thailand did not infect the 25 Nepalese *A. baumannii* clinical isolates.

**Table 1:** List of bacteriophages used in this study.

Previous bacteriophage nomenclature	Bacteriophages (full name; abbreviated name)	Source of isolate	* A. baumannii* host strain	References
ØABP-01; PAB01	vB_AbaP_PhT01; vPhT01	Waste water HE	A1589^a^	Kitti^33^
ØABP-02; PAB02	vB_AbaM_PhT02; vPhT02	Waste water HE	A1389^a^, AB183^b^	Kitti^9,33^
ØABP-04; PAB04	vB_AbaM_PhT04; vPhT04	Waste water HE	A1522^a^, AB22^b^	Kitti^33^
ØABP-05; PAB05	vB_AbaX_PhT05; vPhT05	Waste water HE	A1521^a^	Kitti^9^
ØABP-09; PAB09	vB_AbaX_PhT09; vPhT09	Waste water HE	A1589^a^, AB20^b^	Kitti^33^
ØABP-19; PAB19	vB_AbaP_PhT19; vPhT19	Waste water HE	A1589^a^	Kitti^9,33^
ØABP-25; PAB25	vB_AbaX_PhT25; vPhT25	Waste water HE	A1589^a^	Kitti^33^
ØABP-29; PAB29	vB_AbaP_PhT29; vPhT29	Waste water HG	A1589^a^, AB20^b^	Kitti^9,33^
ØABP-35; PAB35	vB_AbaX_PhT35; vPhT35	Waste water HG	A1589^a^	Kitti^33^
ØABP-38; PAB38	vB_AbaX_PhT38; vPhT38	Waste water HE	A1589^a^	Kitti^33^
ØABP-39; PAB39	vB_AbaP_PhT39; vPhT39	Waste water HE	A1511^a^, AB22^b^	Kitti^9,33^
ØABP-40; PAB40	vB_AbaX_PhT40; vPhT40	Waste water HE	A1522^a^	Kitti^33^
ØABP-44; PAB44	vB_AbaM_PhT44; vPhT44	Waste water HE	ATCC19606^a^, AB20^b^	Kitti^9,33^
ØABP-48; PAB48	vB_AbaX_PhT48; vPhT48	Waste water HG	A1522^a^	Kitti^33^
ØABP-49; PAB49	vB_AbaX_PhT49; vPhT49	Waste water HG	A1521^a^	Kitti^33^
ØABP-52; PAB52	vB_AbaX_PhT52; vPhT52	Waste water HG	A1522^a^	Kitti^33^
ØABP-55; PAB55	vB_AbaX_PhT55; vPhT55	Waste water HG	A1589^a^	Kitti^33^

All phages were isolated in 2010 from Buddhachinaraj hospital (HE) and Bang Rakam hospital (HG), Phitsanulok, Thailand.

^a^Host strains for phage isolation.

^b^Host strains for phage propagation.

### Association between antibiotics, drug resistance patterns, biofilm formation, REP-PCR typing, copper tolerance, and phage susceptibility

Comparisons of phage susceptibility between antibiotics and drug resistance patterns are shown in Table 2. Phage susceptibility was positively associated with resistance to amikacin, ciprofloxacin, cefotaxime, ceftazidime, cefoperazone/sulbactam, imipenem, meropenem, piperacillin/tazobactam, tetracycline, ceftriaxone, cefepime and gentamicin (*p*-value < 0.05). We found the association of phage susceptibility and drug resistance across the MDR-AB, CR-AB, XDR-AB and non MDR-AB strains (*p*-value < 0.05). An analysis of phage susceptibility and biofilm formation also showed association (Table 3). The most common virulence gene associated with biofilm formation, *ompA,* was detected in 94.39% (101/107) of phage susceptible strains (Supplementary Table S1). We found the association of phage susceptibility and present of *ompA* gene (*p*-value < 0.05) (Table3). A repetitive element palindromic-PCR (REP-PCR) analysis of all isolates characterized the phage susceptible *A. baumannii* into 15 REP-types, while phage resistant *A. baumannii* belonged to 22 REP-types. The majority REP-types that presented in bacteriophage susceptible strains were R16 (59/107, 55.14%), R4 (18/107, 16.82%) and R24 (9/107, 8.41%), while the majority REP-types that presented in phage resistant strains were R16 (28/123, 22.76%), R12 (23/123, 18.70%) and R1 (10/123, 8.13%) (Supplementary Table S2). Our results showed the relationships between phage susceptibility and the presence of copper tolerance phenotype and genotype (Table 3).

**Table 2:** Association between antibiotics, drug resistance patterns and phage susceptibility.

Antibiotics/drug resistance patterns	No. of phage susceptible isolates		*p*-values*
	Drug resistant	Drug susceptible	
**Antibiotics**			
Amikacin (AK)	80/123 (65.04%)	27/107 (25.23%)	** < 0.001**
Ciprofloxacin (CIP)	104/196 (53.06%)	3/34 (8.82%)	** < 0.001**
Trimethoprim/sulfamethoxazole (SXT)	75/151 (49.67%)	32/79 (40.51%)	0.1859
Cefotaxime (CTX)	96/185 (51.89%)	11/45 (24.44%)	**0.0009**
Ceftazidime (CAZ)	100/191 (52.35%)	7/39 (17.95%)	**0.0001**
Cefoperazone/sulbactam (CSL)	38/66 (57.57%)	69/164 (42.07%)	**0.0330**
Imipenem (IPM)	103/189 (54.49%)	4/41 (9.76%)	** < 0.001**
Meropenem (MEM)	104/188 (55.32%)	3/42 (7.14%)	** < 0.001**
Piperacillin/tazobactam (PIP)	101/190 (53.16%)	6/40 (15.00%)	** < 0.001**
Tetracycline (TE)	89/142 (62.67%)	18/88 (20.45%)	** < 0.001**
Ceftriaxone (CRO)	101/193(52.33%)	6/37 (16.22%)	**0.0001**
Cefepime (FEP)	89/170 (52.35%)	18/60 (30.00%)	**0.0028**
Gentamicin (CN)	83/146 (56.85%)	24/84 (28.57%)	** < 0.001**
**Drug resistance patterns**			
MDR-AB	105/199 (52.76%)	2/31 (6.45%)	** < 0.001**
CR-AB	103/192 (53.64%)	4/38 (10.53%)	** < 0.001**
XDR-AB	20/28 (71.43%)	87/202 (43.07%)	**0.0048**
Non MDR-AB	2/28 (7.14%)	105/202 (51.98%)	** < 0.001**

*p-values less than 0.05 were considered as a statistically significant difference (Fisher's exact test). Bold font indicates statistically significant difference between two groups.

**Table 3:** Association between biofilm formation, *ompA* gene and copper tolerance among phage susceptible *A. baumannii* strains.

Biofilm formation and copper tolerance	No. of phage susceptible isolates		*p*-values*
	Positive (Yes)	Negative (No)	
**Biofilm formation**			
Biofilm formation phenotype	88/173 (50.87%)	19/57 (33.33%)	**0.0214**
*ompA* gene	101/198 (51.01%)	6/32 (18.75%)	**0.0007**
**Copper tolerance**			
Copper tolerance phenotype	9/53 (16.98%)	98/177 (55.37%)	** < 0.001**
*copRS* gene	9/46 (19.56%)	98/184 (53.26%)	** < 0.001**

*p-values less than 0.05 were considered as a statistically significant difference (Fisher's exact test) and are shown in bold.

### Association between CRISPR-associated (*cas*) genes and phage susceptibility

Across the 230 strains, amplicons of *cas1* (506 bp), *cas2* (196 bp) and *cas3* (850 bp) genes were present in 32 (13.91%), 2 (0.87%) and 30 (13.04%) isolates, respectively. The *cas5* gene was found in 34 (14.78%) isolates, while *cas6* was detected in 7 (3.04%) isolates. The *cas9* gene was not found among any of the *A. baumannii* isolates. Overall, a total of 44 of the 230 strains were found to contain at least one *cas* gene. The majority (31/44, 70.5%) of the *cas* positive strains were classified as phage resistant strains. The correlation between the presence of *cas* genes and phage susceptibility was statistically determined (*p*-value < 0.05), showing that only *cas5* gene was associated with *A. baumannii* phage resistance (Table 4). Phage resistant *A. baumannii* isolates were more than twice as likely to carry two or more *cas* genes than phage susceptible isolates (resistant; 23/123 (18.70%), susceptible; 9/107 (8.41%),) (*p*-value < 0.05) (Table 4).

**Table 4:** Comparisons of CRISPR-associated (*cas*) genes between phage susceptible and resistant *A. baumannii.*

Characteristics	No. of isolates		*p*-values*
	Phage susceptible (n = 107)	Phage resistant (n = 123)	
*cas1* positive	11/107 (10.28%)	21/123 (17.07%)	0.1376
*cas2* positive	0/107 (0.00%)	2/123 (1.67%)	NC
*cas3* positive	9/107 (8.41%)	21/123 (17.07%)	0.0517
*cas5* positive	8/107 (7.47%)	26/123 (21.14%)	**0.0036**
*cas6* positive	2/107 (1.87%)	5/123 (4.06%)	NC
*cas9* positive	0/107 (0.00%)	0/123 (0.00%)	NC
Positive for two or three *cas*-types	9/107 (8.41%)	23/123 (18.70%)	**0.0245**

Bold font indicates statistically significant difference between two groups.

*NC* not comparable.

*p-values less than 0.05 were considered as statistically significant difference (Fisher's exact test).

### Genomic sequence analysis of phage susceptible *A. baumannii*

**Figure 2:**
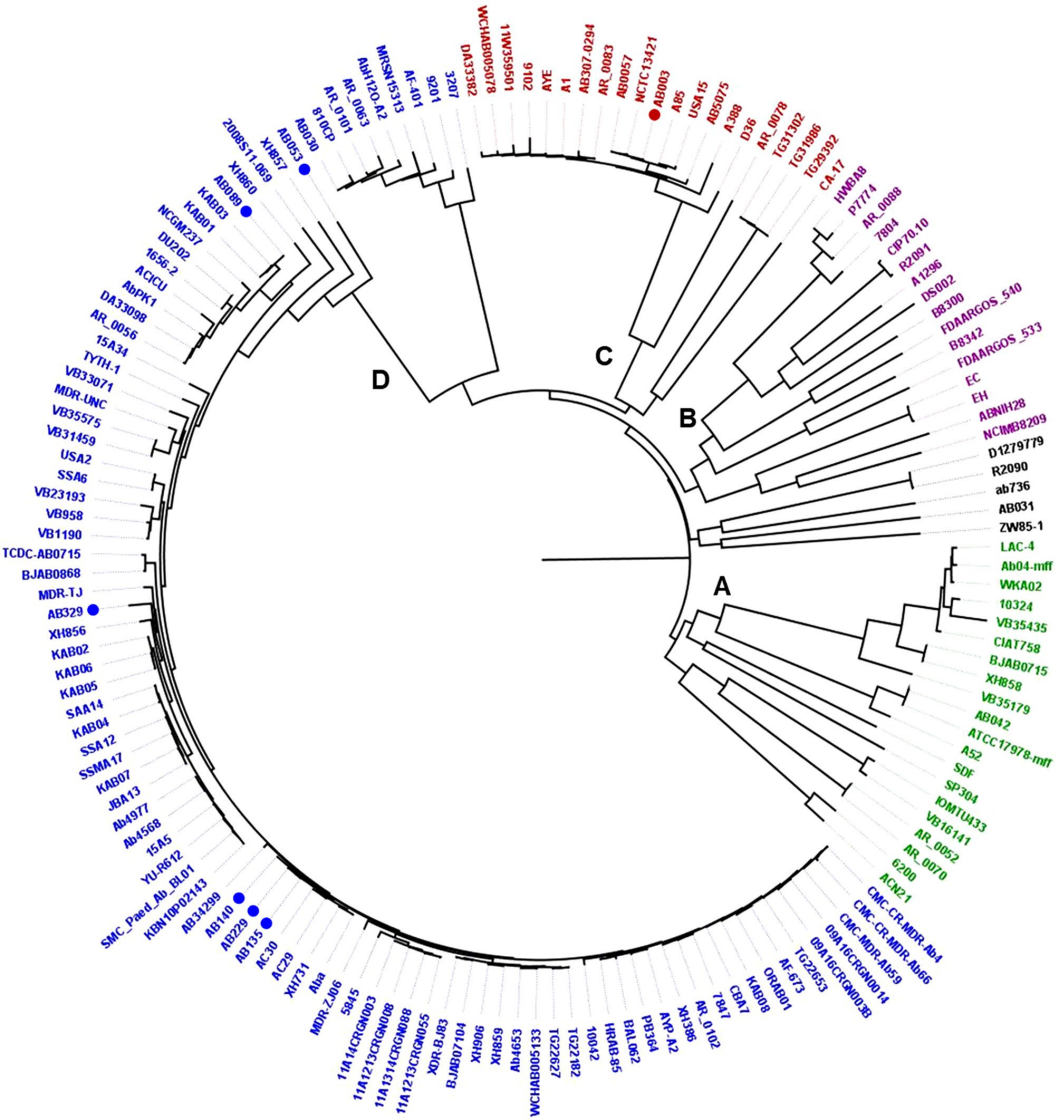
Whole-genome SNP-based phylogenetic tree. A phylogenetic tree base on WGS analysis showing the relationship between seven phage susceptible *A. baumannii* and 149 published *A. baumannii* genomes. Colours indicate four major clusters **(A** = green, **B** = purple, **C** = red, and **D** = blue**)**. Seven phage susceptible *A. baumannii* strains are marked with dots.

The whole genome of seven phage susceptible *A. baumannii* strains (AB003, AB053, AB089, AB135, AB140, AB229 and AB329) was sequenced and the result of the genomic sequence analyses are shown in Fig. 2 and Table 5. Antibiotic resistance genes carried by all phage susceptible *A. baumannii* strains were classified into six groups: sulphonamide (*sul1*, *sul2*), tetracycline (*tet*(B)), β-lactam (*bla*_ADC_, *bla*_OXA_, *bla*_TEM_, *bla*_CARB_), aminoglycoside (*aac(6′)-Ib3, aadA1, ant(2′')-Ia, aph(3′′)-Ib, aph(3′)-Ia, aph(3′)-IIa, aph(3′)-VIa aph(6)-Id, armA*), macrolide (*mph*(E), *msr*(E)), and phenicol (*catB8*). Strain AB003, which is highly phage susceptible (9/17, 53%), carried antibiotic resistance genes in two groups: β-lactam and aminoglycoside. Tetracycline, β-lactam, aminoglycoside, and macrolide resistance genes were detected in most phage susceptible *A. baumannii* (6/7). Multilocus sequence typing (MLST) types 1, 2, 98, and 129 (Pasteur) were investigated in phage susceptible *A. baumannii*. Two strains (AB003 and AB053) carried one complete prophage each and had acquired copper tolerance genes. Two complete predicted prophages contained genome size ranging from 31.3 to 40.6 kb and GC contents ranging from 39.10 to 40.01%. Incomplete prophage regions were detected in all seven strains. All seven strains carried biofilm associated genes including *ompA, adeRS, csuE, gacS, csuCD, bap,* and *bfmS* genes. Plasmid groups, GR1 (1/7), GR2 (7/7), GR6 (4/7), and pRAY (1/7) were detected (Table 5). CRISPR-associated (*cas*) genes were only detected in two strains, AB003 and AB329, when searched by CRISPRCasFinder. The creation of a whole-genome SNP-based phylogenetic tree using the seven *A. baumannii* genomes and 149 published *A. baumannii* genomes indicated that these bacteria are divided into four large clusters, which are designated as clusters A, B, C, and D (Fig. 2). Strain AB003 was classified into cluster C. Other phage susceptible *A. baumannii* strains from this study were grouped into cluster D (Fig. 2).

**Table 5:** Genome features, acquired antimicrobial resistance genes, putative prophages, copper tolerance, virulence genes, and CRISPR-associated (*cas*) genes identified in whole genome sequences of phage susceptible *A. baumannii.*

Strain ID/Genome characteristics	AB003	AB053	AB089	AB135	AB140	AB229	AB329
Hospital/year isolated	HE/2006	HA/2013	HA/2013	HB/2014	HB/2014	HB/2014	HD/2015
Specimens	Sputum	Sputum	Sputum	Pus	Sputum	Urine	Sputum
Phage susceptibility	(9/17)	(2/17)	(3/17)	(3/17)	(4/17)	(3/17)	(6/17)
**Genome features**							
Number of assembled contigs	57	142	174	99	77	333	256
Average genome size (bp)	4,050,570	4,108,360	3,897,375	4,036,789	3,990,813	4,070,648	3,943,615
GC content (%)	38.8	38.9	39.0	38.9	39.8	39.1	39.0
Number of CDSs	3,965	4,059	3,808	3,955	3,875	4,134	3,923
Number of RNAs	70	69	70	72	73	68	68
MLST/Rep type	ST1/R34	ST129/R15	ST2/R4	ST2/R4	ST2/R4	ST2/R4	ST98/R4
**Acquired antimicrobial resistance genes**							
Sulfonamide resistance	ND	*sul1*, *sul1*	*sul2*	*sul2*	ND	*sul2*	*sul2*
Tetracycline resistance	ND	*tet(B)*	*tet(B)*	*tet(B)*	*tet(B)*	*tet(B)*	*tet(B)*
Beta-lactam resistance	*bla*_*ADC-25*_*, bla*_*OXA-23*_*, bla*_*OXA-69*_*, bla*_*TEM-181*_	*bla*_ADC-25_, *bla*_OXA-23_, *bla*_OXA-66_	*bla*_ADC-25_, *bla*_OXA-23_, *bla*_OXA-66_, *bla*_TEM-1D_	*bla*_ADC-25_, *bla*_OXA-23_, *bla*_OXA-66_, *bla*_TEM-1D_	*bla*_ADC-25_, *bla*_CARB-16/49/5_, *bla*_OXA-23_, *bla*_OXA-66_, *bla*_TEM-1D_	*bla*_ADC-25_, *bla*_OXA-23_, *bla*_OXA-66_, *bla*_TEM-1D_	*bla*_ADC-25_, *bla*_OXA-23_, *bla*_OXA-66_, *bla*_TEM-1D_
Aminoglycoside resistance	*aph(3′)-IIa*	*aac(6′)-Ib3, aadA1, ant(2′')-Ia, aph(3′')-Ib, aph(3′)-Ia, aph(6)-Id, armA*	*aph(3′')-Ib, aph(3′)-Ia, aph(6)-Id, armA*	*aph(3′')-Ib, aph(3′)-Ia, aph(6)-Id, armA*	*aph(3′')-Ib, aph(3′)-*VIa*, aph(6)-Id, armA*	*aph(3′')-Ib, aph(3′)-Ia, aph(6)-Id, armA*	*aph(3′')-Ib, aph(3′)-Ia, aph(6)-Id, armA*
Macrolide resistance	ND	*mph(E), msr(E)*	*mph(E), msr(E)*	*mph(E), msr(E)*	*mph(E), msr(E)*	*mph(E), msr(E)*	*mph(E), msr(E)*
Phenicol resistance	ND	*catB8*	ND	ND	ND	ND	ND
**Virulence genes**							
Iron acquisition	*entE*, *zur*	*entE*, *zur*	*entE*, *zur*	*entE*, *zur*	*entE*, *zur*	*entE*, *zur*	*entE*, *zur*
Biofilm formation	*ompA, adeRS, csuE, gacS, csuCD, bfmS*	*ompA, adeRS, gacS, bfmS*	*ompA, adeRS, csuE, gacS, csuCD, bap, bfmS*	*ompA, adeRS, csuE, gacS, csuCD, bap, bfmS*	*ompA, csuE, gacS, csuCD, bap, bfmS*	*ompA, adeRS, csuE, gacS, csuCD, bap, bfmS*	*ompA, adeRS, csuE, gacS, csuCD, bfmS*
Type V, VI and IV secretion systems	*hcp, traU, traC*	*ata*, *hcp*	*ata*, *hcp*	*ata*, *hcp, traU, traC*	*ata*, *hcp, traU, traC*	*ata*, *hcp, traU, traC*	*ata*, *hcp, traU, traC*
Other systems	*ostA*, *ompF*, *rstA*	*ostA*, *ompF*, *rstA*	*ostA*, *ompF*, *rstA*	*ostA*, *ompF*, *rstA*	*ostA*, *ompF*, *rstA*	*ostA*, *ompF*, *rstA*	*ostA*, *ompF*, *rstA*
Copper tolerance (genotype/phenotype)	*copRS*+/tolerance	*copRS*+/tolerance	*copRS−/*susceptible	*copRS−/*susceptible	*copRS−/*susceptible	*copRS−/*susceptible	*copRS−/*susceptible
Number of prophages (complete/incomplete)	9 (1/8)	8 (1/7)	4 (0/4)	10 (0/10)	5 (0/5)	8 (0/8)	6 (0/6)
Plasmid replicon typing (GR)	GR2, GR6	GR1, GR2, pRAY	GR2	GR2, GR6	GR2, GR6	GR2, GR6	GR2
CRISPR-associated (*cas*) genes	CAS-TypeI (*cas6, cas3-cas2, cas1*)	Negative	Negative	Negative	Negative	Negative	CAS-TypeI (*cas3*)
Accession no/Bioproject	PRJEB32181	JABCNM000000000	JABCNJ000000000	JABCNK000000000	JABCNI000000000	JABCNL000000000	JABCNH000000000
Reference	Thummeepak et al.^35^	This study	This study	This study	This study	This study	This study

*ND* not detected.

## Discussion

In Thailand, the incidence of MDR-AB, CR-AB and XDR-AB infection has increased in the past decade^3,11^. Tigecycline and colistin are still last resort drugs of choice for treatment of multidrug-resistant *A. baumannii*. However, cases of colistin resistance in *A. baumannii* was reported in Thailand and can cause serious problems in treatment outcome^12^. Phage therapy is one potential candidate for the treatment of multidrug resistant bacteria. We found that 53.5% of *A. baumannii* strains isolated from six hospitals in Thailand and Nepal were resistant to all 17 phages tested. This is close to what was found previously by Thawal et al. in 2012, where approximately 51% of *A. baumannii* strains tested in this study were found to be phage resistant^13^. We found that *A. baumannii* strains were more sensitive to phages from the same geographic area, since the Thai phages could not lyse *A. baumannii* strains isolated from Nepal. This is also consistent with previously published investigation, where one study has reported that phage-host specificity was limited to specific geographic areas^14^. Host strain AB003, which was highly susceptible to bacteriophages, was isolated from the same hospital (HE), where the phages of this study were isolated. Most of the 26 non MDR-AB (92.8%) strains were classified as phage resistance. Our finding is consistent with previous studies by Kitti et al. and Chen et al. that showed that antibiotic-resistant *A. baumannii* clinical isolates have higher susceptibility to phages than antibiotic-sensitive strains^9,10^. The increase in phage susceptibility may be a result of the high antibiotic usage in the hospital environment and co-evolution of phages and bacteria^10^. Chen et al. proposed that the antibiotic-resistant *A. baumannii* strains that remained in the hospital could be more easily infected by phages, because of the adaptations for multiple antibiotics resistance, especially in medical environments that have high antibiotic use since the phage resistant strains of *A. baumannii* displayed diverse RAPD-PCR types, when compared with phage susceptible strains^10^. Our study found that phage resistant strains of *A. baumannii* can be classified into more than 20 different REP-types, while 80% of phage susceptible strains can be grouped into just three similar REP-types (R4, R16 and R24). The phage resistant strains were derived from various sources and did not have a common stressor or environmental selector meaning they presented a diversity of REP-type patterns. In contrast, the phage susceptible strains that can infect by at least one phage may be caused by the selective pressure of adopting resistance to multiple antibiotics and limitation of phage immunity.

Bacterial biofilm formation is one strategy mediating protection against phage lysis. However, our study showed positive association between biofilm formation and phage susceptibility. Interestingly, some studies also revealed that phages can induce and strengthen biofilms and it has been shown that biofilm composition and maturation had an impact on susceptibility towards phage infection^15^. Quorum sensing system, OmpA and OmpC are critical host factors for phage infection^7,16,17^. The receptor for *Shigella flexneri* phage Sf6 and *E. coli* phage M1 is the outer membrane protein A **(**OmpA**).** In this study, 94.39% (101/107) of phage susceptible strains *A. baumannii* harbored the *ompA* gene. We found positive association between the present of OmpA in phage susceptible strains (Table3). These data imply that OmpA could also be a receptor for *A. baumannii* phage adsorption.

Bacteria have numerous strategies to prevent bacteriophage infection and cell lysis. The CRISPR/Cas systems are one of the phage immunity systems that are present in bacteria and enabling the organisms to respond to and eliminate invading genetic material^18^. In 2016 Lin et al*.* showed that *Klebsiella pneumoniae* isolated from the hospital environment have a decreased level of phage immunity and a reduction in CRISPR-Cas activity compared to strains isolated from outside the hospital environment^19^. PCR with *cas*-specific primers showed that 19% of the *A. baumannii* isolates had a CRISPR-Cas system. The majority (70.5%) of the *cas* positive strains were classified as phage resistant strains. The positive correlation between the presence of *cas5* or combinations of two or three different *cas* genes and phage resistant strains was statistically determined (*p*-value < 0.05). This data supports a hypothesis that CRISPR/Cas systems are one of the important phage resistance mechanisms in *A. baumannii.* We analyzed whole genome sequence of *Acinetobacter* spp. deposited on National Center for Biotechnology Information (NCBI) database showed that these contain six *cas* genes including *cas*1*, cas*2*, cas*3*, cas*5*, cas*6 and *cas*9^20,21^. Cas1 and Cas2 proteins recognize invading phage nucleic acids and insert them into the CRISPR/Cas array as a spacer in order to transcribe into pre-CRISPR-RNA (crRNA)^22^, whereas, the Cas5 protein has RNase activity, resulting in inhibition of the transcription machinery in phages^18^. The *cas*6 gene encodes a type I-F CRISPR-associated endoribonuclease Cas6/Csy4 protein, which has an important role in the cleavage of the repeat sequences to yield mature CRISPR-RNAs (crRNAs) that complementary pair with invading phage nucleic acid bases, causing destruction of the target phage DNA^23^.

Phage resistance may be related to lower virulence, making the resistant bacteria less virulent than non-phage-resistant strains^24^. We detected antibiotic resistance, CRISPR-associated (*cas*) genes and virulence genes among *A. baumannii* isolates using PCR and found association between phage susceptibility with antibiotic resistance, CRISPR-associated (*cas*) genes and virulence genes. Whole genome sequencing of virulence genes involving biofilm formation revealed that the seven phage susceptible *A. baumannii* in this study harbored various virulence genes linked to the ability to form biofilms, iron acquisition and bacterial secretion systems. *cas* genes (*cas1, cas3-cas2,* and *cas6*) were found in two of seven the phage susceptible *A. baumannii* genomes (AB003 and AB329). Antibiotic resistance genes involved sulphonamide, tetracycline, β-lactam, aminoglycoside, macrolide and phenicol resistance were found in seven phage susceptible *A. baumannii* genomes. In a previous study, we identified the acquired copper tolerance genes, *copRS* that respond to copper toxicity in the genome of *A. baumannii*^25^. These genes were identified in two of the seven phage susceptible strains and both strains also showed the copper tolerance phenotype. The correlation between phage susceptibility and regulating heavy metal toxicity is in agreement with the findings of Zhang et al., where plasmid-borne cadmium resistant determinants were associated with the susceptibility of *Listeria monocytogenes* to phages^26^. Antibiotic resistance genes and heavy metal tolerance genes can be disseminated within the microbial population by horizontal gene transfer mechanisms using plasmids and phages. Among 19 groups of plasmids identified in *A. baumannii*, in this study, we found plasmids GR1, GR2, GR6, and pRAY in phage susceptible *A. baumannii.* Plasmid GR6, linked to the dissemination via horizontal gene transfer by conjugation was detected in four strains of phage susceptible *A. baumannii*^27^*.* In contrast, phages are important genetic vehicles for transferring genetic information between bacteria via transduction^28^. All seven phage susceptible *A. baumannii* strains in our analysis carried prophage associated sequences on their chromosomes. However, only two strains harbored complete prophages. Prophages are directly related to genome diversity, evolution and strains variation as well as an association with the presence of antibiotic resistance genes and virulence genes^29,30^. In addition, prophages are responsible for gene disruption or translocation to phenotypic changes in their host and can introduction of pathogenicity determinants that contribute positively to bacterial fitness^29,31^.

An analysis of the clonal relationship of the seven phage susceptible *A. baumannii* genomes from this study with 149 previously published *A. baumannii* genomes (Fig. 2.) revealed that strain AB003, isolated from hospital HE, was closely related to strains AB0057 (CP001182), NCTC13421 (LS483472), A85 (CP021782) and USA15 (CP020595). The three strains isolated from hospital HB **(**AB140, AB229 and AB135**)** all belonged to the same ST type and are very closely related with Malaysian and Chinese isolates AC30 (CP007577), AC29 (CP007535), XH731 (CP021321) and Aba (CP030083). The presence of closely related bacterial strains collected from Thailand, Malaysia and China might be descriptive that these organisms shared ancestry and emerged at the same time, caused by rapid dissemination of genetic material.

In conclusion, we found that phage susceptibility was associated with antibiotic resistance and virulence among *A. baumannii* strains. Moreover, in silico analysis showed that seven strains of phage susceptible *A. baumannii* carried at least six antibiotic resistance genes which included sulphonamide, tetracycline, β-lactam, aminoglycoside, macrolide, phenicol and several virulence genes involved biofilm formation. Thus, the data from this study can be used as essential information about phage therapy in the future.

## Materials and methods

### Bacteria and phages used in this study

We used 230 *A. baumannii* isolates collected from inpatient units of five hospitals in Thailand (HA-HE) and one hospital in Nepal (HF) as described by Niumsup et al., Joshi et al., and Leungtongkam et al.^2,3,32^ (Supplementary Table S1). Seventeen *A. baumannii* phages used in this study were isolated from wastewater treatment plants of two hospitals (HE and HG) in Phitsanulok province, Thailand^9,33^ (Table 1). *A. baumannii* strains used as the host for phage propagation are shown in Table 1. The protocol was approved by Naresaun University Institutional Biosafety Committee (NUIBC) (No. NUIBC GM 62-06-22).

### Antimicrobial susceptibility testing

Antimicrobial susceptibility testing was performed using disk diffusion method according to the Clinical and Laboratory Standards Institute (CLSI) guidelines (2017) with fifteen antibiotics; amikacin, cefepime, cefotaxime, cefoperazone/sulbactam, ceftazidime, ceftriaxone, ciprofloxacin, colistin, gentamicin, imipenem, meropenem, piperacillin/tazobactam, tetracycline, tigecycline and trimethoprim/sulfamethoxazole. *Escherichia coli* ATCC 25922 was used as a quality control strain. All isolates were defined as being MDR-AB, when there was resistance to more than three antibiotic classes, as carbapenem- resistant *A. baumannii* (CR-AB), when there was resistance to carbapenems and as XDR-AB, when there was resistance to all antimicrobial agents tested except the polymyxin colistin, and tigecycline. Non MDR-AB classified as bacteria that non-resistant or less resistant than the three antibiotic group^34^.

### Phages susceptibility of A. baumannii

Assessment of phage susceptibility was determined by spot test on all *A. baumannii* isolates. A colony of bacteria was suspended in 0.85% (w/v) NaCl to the equivalent of a 0.5 McFarland standard (1 × 10^8^ CFU/ml). The suspensions were swabbed on to Trypticase Soy Agar (TSA). Phage suspensions (2 µl at 1 × 10^8^ PFU/ml) were dropped into the bacterial lawn. Then, plates were incubated at 37 °C for 8 h. The result of spot test being clearance at the location of phage inoculation when the host was sensitive to phage (Supplementary Fig. S1). All experiments were performed in duplicate.

### Biofilm formation, detection of ompA gene, repetitive element palindromic-PCR (REP-PCR) and copper tolerance

The genetic diversity among phage susceptible *A. baumannii* isolates was studied using REP-PCR typing as described previously by Leungtongkam et al.^3^. Detection of *ompA* gene and biofilm formation were performed as described by Thummeepak et al.^35^. The copper tolerance phenotype and copper-related genes (*copRS*) studies were conducted by using Minimum Inhibitory Concentration (MIC) and PCR methods as described previously^25^.

### Detection of CRISPR-associated (cas) genes

The bacterial protein sequences and GenBank nucleotide sequences of well-identified *cas* genes, detected in six example *Acinetobacter* spp., were used as templates to design the specific primers using Primer-BLAST program (https://www.ncbi.nlm.nih.gov/tools/primer-blast/) (Supplementary Table S3). The genomic DNA of all isolates in this study was extracted by a previously described boiling method^36^ for use as a template in a multiplex-PCR method with *cas* specific primers (Supplementary Table S3) to detect CRISPR-associated (*cas*) genes. Conditions for multiplex-1 was the following: 95 °C for 5 min, and then 35 cycles at 94 °C for 30 s, at 62 °C for 40 s, and 72 °C for 50 s, followed by a final extension at 72 °C for 5 min. Multiplex-2 condition was one cycle of 95 °C for 5 min, followed by 35 cycles at 94 °C for 30 s, 58 °C for 40 s, and 72 °C for 50 s and finally, one cycle of 72 °C for 5 min. The PCR product was visualized by agarose gel electrophoresis, stained with ethidium bromide in a UV transilluminator.

### Whole genome sequencing of A. baumannii

We selected seven phage susceptible *A. baumannii* strains (Group1 and Group2) that were susceptible to phages from all four clusters, namely AB003 (cluster III), AB053 (cluster IV), AB089 (cluster I), AB135 (cluster I), AB140 (cluster II), AB229 (cluster I) and AB329 (cluster IV) to study the whole genome sequence. Genomic DNA of *A. baumannii* strains were isolated using HiYield Genomic DNA Mini Kit (RBC Bioscience, New Taipei, Taiwan). Extracted DNA was quantified using a Qubit DNA Assay Kit in a Qubit 2.0 Fluorometer (Life Technologies, CA, USA). Genomes were sequenced by the Illumina Miseq platform (250 bp paired end). DNA libraries were constructed by Nextera XT DNA Library Preparation Kit according to the manufacturers’ instructions. Reads were trimmed and assembled by using Sickle v1.33^37^ and SPAdes genome assembler v3.6.0^38^ with default settings, respectively. After assembled contigs, annotation was conducted with RAST pipeline using default parameters^39^. Single nucleotide polymorphisms (SNPs) phylogenetic analysis was conducted by using CSI Phylogeny v1.4 with default options^40^ (with reference strain SDF, NC_010400.1). Phylogenetic tree image was visualized and edited by FigTree v1.4.4 (https://tree.bio.ed.ac.uk/software/figtree/). Antibiotic resistance genes in draft genomes were detected by ResFinder tool^41^. CRISPR-associated (*cas*) genes within bacterial genomes were detected by using CRISPRCasFinder programs and the prediction of prophage regions genomes were performed by using PHASTER^42,43^. Prophage hits with status “questionable” were scored as incomplete prophages. The virulence genes were examined by BlastN algorithm using collection of virulence genes as queries.

### Statistical analysis

The Fisher’s exact test was used to analyze phage susceptibility associated with the drug resistance patterns, biofilm formation, *ompA* gene, copper tolerance and CRISPR-associated (*cas*) genes. Data with a *p*-value < 0.05 were classed as statistically significant.

### Nucleotide sequence accession number

The nucleotide sequences of seven *A. baumannii* have been deposited to the GenBank database under accession numbers PRJEB32181 (AB003), JABCNM000000000 (AB053), JABCNJ000000000 (AB089), JABCNK000000000 (AB135), JABCNI000000000 (AB140), JABCNL000000000 (AB229), and JABCNH000000000 (AB329).

## Supplementary information

Supplementary Information.

## References

[1] Tal-Jasper R (2016). Clinical and epidemiological significance of carbapenem resistance in *Acinetobacter baumannii* infections. Antimicrob. Agents Chemother..

[2] Joshi PR (2017). Co-existence of *bla*_OXA-23_ and *bla*_NDM-1_ genes of *Acinetobacter baumannii* isolated from Nepal: antimicrobial resistance and clinical significance. Antimicrob. Resist. Infect. Control..

[3] Leungtongkam U (2018). Dissemination of *bla*_OXA-23_, *bla*_OXA-24_, *bla*_OXA-58_, and *bla*_NDM-1_ genes of *Acinetobacter baumannii* isolates from four tertiary hospitals in Thailand. Microb. Drug Resist..

[4] Loc-Carrillo C, Abedon ST (2011). Pros and cons of phage therapy. Bacteriophage.

[5] Bertozzi Silva J, Storms Z, Sauvageau D (2016). Host receptors for bacteriophage adsorption. FEMS Microbiol. Lett..

[6] Morona R, Krämer C, Henning U (1985). Bacteriophage receptor area of outer membrane protein OmpA of *Escherichia coli* K-12. J. Bacteriol..

[7] Parent KN (2014). OmpA and OmpC are critical host factors for bacteriophage Sf6 entry in Shigella. Mol. Microbiol..

[8] Azam AH, Tanji Y (2019). Bacteriophage-host arm race: An update on the mechanism of phage resistance in bacteria and revenge of the phage with the perspective for phage therapy. Appl. Microbiol. Biotechnol..

[9] Kitti T, Thummeepak R, Leungtongkam U, Kunthalert D, Sitthisak S (2015). Efficacy of *Acinetobacter baumannii* bacteriophage cocktail on *Acinetobacter baumannii* growth. Afr. J. Microbiol. Res..

[10] Chen LK (2017). Clinical Antibiotic-resistant *Acinetobacter baumannii* strains with higher susceptibility to environmental phages than antibiotic-sensitive strains. Sci. Rep..

[11] Inchai J (2015). Risk factors of multidrug-resistant, extensively drug-resistant and pandrug-resistant *Acinetobacter baumannii* ventilator-associated pneumonia in a Medical Intensive Care Unit of University Hospital in Thailand. J. Infect. Chemother..

[12] Naksena P, Tribuddharat C, Thaipisuttikul I (2012). Colistin resistance in *Acinetobacter baumannii* isolated in Siriraj Hospital is associated with mutations in pmrCAB operon. Am. J. Med..

[13] Thawal ND, Yele AB, Sahu PK, Chopade BA (2012). Effect of a novel podophage AB7-IBB2 on *Acinetobacter baumannii* biofilm. Curr. Microbiol..

[14] Yang Z (2019). Characterization and genome annotation of a newly detected bacteriophage infecting multidrug-resistant *Acinetobacter baumannii*. Arch. Virol..

[15] Hansen MF, Svenningsen SL, Røder HL, Middelboe M, Burmølle M (2019). Big Impact of the Tiny: Bacteriophage-Bacteria Interactions in Biofilms. Trends Microbiol..

[16] Hashemolhosseine S, Holmes Z, Mutschler B, Henning U (1994). Alterations of receptor specificities of coliphages of the T2 family. J. Mol. Biol..

[17] Saucedo-Mora MA (2017). Selection of functional quorum sensing systems by lysogenic bacteriophages in *Pseudomonas aeruginosa*. Front. Microbiol..

[18] Makarova KS (2015). An updated evolutionary classification of CRISPR–Cas systems. Nat. Rev. Microbiol..

[19] Lin TL (2016). Imipenem represses CRISPR-Cas interference of DNA acquisition through H-NS stimulation in *Klebsiella pneumoniae*. Sci. Rep..

[20] Sahl JW (2015). Phylogenetic and genomic diversity in isolates from the globally distributed *Acinetobacter baumannii* ST25 lineage. Sci. Rep..

[21] Nicoloff H, Hjort K, Levin BR, Andersson DI (2019). The high prevalence of antibiotic heteroresistance in pathogenic bacteria is mainly caused by gene amplification. Nat. Microbiol..

[22] Maxwell KL (2016). Phages fight back: inactivation of the CRISPR-Cas bacterial immune system by anti-CRISPR proteins. PLOS Pathog.

[23] Wiedenheft B (2011). RNA-guided complex from a bacterial immune system enhances target recognition through seed sequence interactions. Proc. Natl. Acad. Sci. USA.

[24] León M, Bastías R (2015). Virulence reduction in bacteriophage resistant bacteria. Front. Microbiol..

[25] Thummeepak R (2020). Essential gene clusters involved in copper tolerance identified in *Acinetobacter baumannii* clinical and environmental isolates. Pathogens.

[26] Zhang H (2015). Plasmid-borne cadmium resistant determinants are associated with the susceptibility of *Listeria monocytogenes* to bacteriophage. Microbiol. Res..

[27] Leungtongkam U (2018). Acquisition and transfer of antibiotic resistance genes in association with conjugative plasmid or class 1 integrons of *Acinetobacter baumannii*. PLoS ONE.

[28] Balcazar JL (2014). Bacteriophages as vehicles for antibiotic resistance genes in the environment. PLOS Pathog..

[29] Fortier LC, Sekulovic O (2013). Importance of prophages to evolution and virulence of bacterial pathogens. Virulence..

[30] Brueggemann AB (2017). Pneumococcal prophages are diverse, but not without structure or history. Sci. Rep..

[31] Brüssow H, Canchaya C, Hardt WD (2004). Phages and the evolution of bacterial pathogens: from genomic rearrangements to lysogenic conversion. Microbiol. Mol. Biol. Rev..

[32] Niumsup PR, Boonkerd N, Tansawai U, Tiloklurs M (2009). Carbapenem-resistant *Acinetobacter baumannii* producing OXA-23 in Thailand. Jpn. J. Infect. Dis..

[33] Kitti T (2014). Characterization and detection of endolysin gene from three *Acinetobacter baumannii* bacteriophages isolated from sewage water. Indian J. Microbiol..

[34] Magiorakos AP (2012). Multidrug-resistant, extensively drug-resistant and pandrug-resistant bacteria: an international expert proposal for interim standard definitions for acquired resistance. Clin. Microbiol. Infect..

[35] Thummeepak R, Kongthai P, Leungtongkam U, Sitthisak S (2016). Distribution of virulence genes involved in biofilm formation in multi-drug resistant *Acinetobacter baumannii* clinical isolates. Int. Microbiol..

[36] Dashti AA, Jadaon MM, Abdulsamad AM, Dashti HM (2009). Heat treatment of bacteria: a simple method of DNA extraction for molecular techniques. Kuwait Med J..

[37] Joshi, N. A. & Fass, J. N. *Sickle: A sliding-window, adaptive, quality-based trimming tool for FastQ files (Version 1.33) [Software]*. https://github.com/najoshi/sickle (2011).

[38] Bankevich A (2012). SPAdes: a new genome assembly algorithm and its applications to single-cell sequencing. J. Comput. Biol..

[39] Aziz RK (2008). The RAST Server: rapid annotations using subsystems technology. BMC Genom..

[40] Kaas RS, Leekitcharoenphon P, Aarestrup FM, Lund O (2014). Solving the problem of comparing whole bacterial genomes across different sequencing platforms. PLoS ONE.

[41] Zankari EH (2012). Identification of acquired antimicrobial resistance genes. J. Antimicrob. Chemother..

[42] Couvin D (2018). CRISPRCasFinder, an update of CRISRFinder, includes a portable version, enhanced performance and integrates search for Cas proteins. Nucleic Acids Res..

[43] Arndt D (2016). PHASTER: a better, faster version of the PHAST phage search tool. Nucleic Acids Res..

