# Supplementary material for: Genomic analysis reveals high virulence and antibiotic resistance amongst phage susceptible Acinetobacter baumannii
Source: Sci Rep. 2020 Sep 30;10:16154. doi: 10.1038/s41598-020-73123-y (PMC7528101; doi:10.1038/s41598-020-73123-y)
Supplement: Supplementary file 1 — Supplementary Information. [file 41598_2020_73123_MOESM1_ESM.pdf]

Genomic analysis reveals high virulence and antibiotic resistance amongst phage susceptible *Acinetobacter baumannii*

Udomluk Leungtongkam[1], Rapee Thummeepak[1], Thawatchai Kitti[2], Kannipa Tasanapak[1], Jintana Wongwigkarn[1], Kathryn M. Styles[3], Elizabeth M. H. Wellington[3], Andrew D. Millard[4] Antonia P. Sagona[3], and Sutthirat Sitthisak[1*]

[1]Department of Microbiology and Parasitology, Faculty of Medical Science, Naresuan University, Phitsanulok, 65000, Thailand

[2]Faculty of Oriental Medicine, Chiang Rai College, Chiang Rai, 57000, Thailand

[3]School of Life Sciences, University of Warwick, Coventry, CV4 7AL, United Kingdom

[4]Department of Genetics and Genome Biology, University of Leicester, Leicester, LE1 7RH, United Kingdom

*Correspondence: Associate Professor Dr. Sutthirat Sitthisak

Address: Department of Microbiology and Parasitology, Faculty of Medical Science, Naresuan University, Phitsanulok, Thailand

(Tel): 66-55-964626, 66-84-5734203

(Fax): 66-55-964770

E-mail: sutthirats@nu.ac.th

Fig. S1 Bacteriophage susceptibility test of vPhT02, vPhT04, vPhT39 and vPhT40 on *A. baumannii* strain AB329.

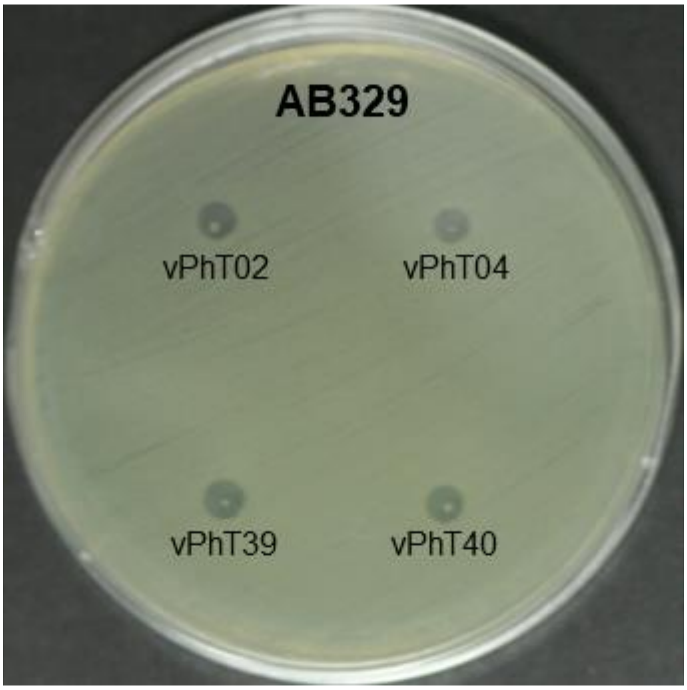

Table S1. Supplement of 230 *A. baumannii* isolates

No	Strain ID	Hospitals	Phage susceptibility	Rep-types	Antibiotic resistance[a]	Phenotype	*ompA* gene	Biofilm formation	CRISPR-associated (*cas*) genes
1	AB002	HE	0	R34	AK/CIP/SXT/CTX/IPM/MEM/PIP/TE/CRO/CN	MDR-AB, CR-AB	+	-	-
2	AB003	HE	9	R34	CIP/CTX/CAZ/IPM/MEM/PIP/CRO	MDR-AB, CR-AB	+	+	*cas1, cas3, cas5*
3	AB004	HE	0	R34	CIP/CTX/CAZ/IPM/MEM/PIP/CRO/CN	MDR-AB, CR-AB	+	+	*cas1, cas3, cas5*
4	AB005	HE	0	R34	CIP/CTX/CAZ/IPM/MEM/PIP/CRO/CN	MDR-AB, CR-AB	+	-	*cas1, cas3, cas5*
5	AB007	HE	0	R34	AK/CIP/SXT/CTX/CAZ/IPM/MEM/PIP/CRO/FEP/CN	MDR-AB, CR-AB	+	-	-
6	AB008	HE	4	R34	AK/CIP/SXT/CTX/IPM/MEM/PIP/TE/CRO/CN	MDR-AB, CR-AB	+	-	-
7	AB009	HE	0	R34	AK/CIP/SXT/CTX/IPM/MEM/PIP/CRO/CN	MDR-AB, CR-AB	-	-	-
8	AB010	HE	0	R34	AK/CIP/SXT/CTX/CAZ/IPM/MEM/PIP/CRO/FEP/CN	MDR-AB, CR-AB	+	-	-
9	AB011	HE	4	R34	AK/CIP/SXT/CTX/IPM/MEM/PIP/TE/CRO/CN	MDR-AB, CR-AB	+	+	-
10	AB012	HE	10	R34	CIP/CTX/CAZ/IPM/MEM/PIP/CRO	MDR-AB, CR-AB	+	+	*cas1, cas3, cas5*
11	AB013	HE	6	R34	AK/CIP/SXT/CTX/CAZ/IPM/MEM/PIP/TE/CRO/FEP/CN	MDR-AB, CR-AB	+	+	-
12	AB014	HA	2	R1	AK/CIP/SXT/CTX/CAZ/IPM/MEM/PIP/TE/FEP/CN	MDR-AB, CR-AB	+	+	-
13	AB015	HA	0	R6	No resistance	Non MDR-AB	-	+	*cas1, cas3, cas5*
14	AB016	HA	2	R4	AK/CIP/SXT/CTX/CAZ/CSL/IPM/MEM/PIP/TE/CRO/FEP/CN	MDR-AB,CR-AB, XDR-AB	+	+	-
15	AB021	HA	0	R1	CIP/SXT/CAZ/IPM/MEM/PIP/TE/CRO/FEP/CN	MDR-AB, CR-AB	+	+	-
16	AB022	HA	4	R4	AK/CIP/SXT/CTX/CAZ/CSL/IPM/MEM/PIP/TE/CRO/FEP/CN	MDR-AB,CR-AB, XDR-AB	+	+	-
17	AB023	HA	0	R16	AK/CIP/SXT/CTX/CAZ/IPM/MEM/PIP/CRO/FEP/CN	MDR-AB, CR-AB	+	+	-
18	AB025	HA	4	R16	AK/CIP/SXT/CTX/CAZ/IPM/MEM/PIP/TE/CRO/FEP/CN	MDR-AB, CR-AB	+	+	-
19	AB027	HA	0	R16	IPM/MEM	CR-AB	+	+	*cas2, cas6*
20	AB028	HA	2	R4	AK/CIP/SXT/CTX/CAZ/CSL/IPM/MEM/PIP/TE/CRO/FEP/CN	MDR-AB,CR-AB, XDR-AB	+	+	-
21	AB029	HA	2	R16	AK/CIP/SXT/CTX/CAZ/IPM/MEM/CRO/FEP/CN	MDR-AB, CR-AB	+	+	-
22	AB030	HA	2	R16	AK/CIP/SXT/CTX/CAZ/CSL/IPM/MEM/PIP/CRO/FEP/CN	MDR-AB, CR-AB	+	+	-
23	AB032	HA	0	R7	No resistance	Non MDR-AB	-	+	-
24	AB035	HA	0	R6	No resistance	Non MDR-AB	-	+	*cas1, cas3, cas5*
25	AB037	HA	0	R8	No resistance	Non MDR-AB	-	+	*cas1, cas3, cas5*
26	AB038	HA	0	R16	AK/CIP/SXT/CTX/CAZ/IPM/MEM/PIP/CRO/FEP/CN	MDR-AB, CR-AB	-	+	*cas5*
27	AB039	HA	0	R9	No resistance	Non MDR-AB	-	+	-
28	AB040	HA	2	R16	AK/CIP/SXT/CTX/CAZ/CSL/IPM/MEM/PIP/CRO/FEP/CN	MDR-AB, CR-AB	+	+	-
29	AB043	HA	2	R16	AK/CIP/CTX/CAZ/CSL/IPM/MEM/PIP/CRO/FEP/CN	MDR-AB, CR-AB	+	+	-
30	AB044	HA	0	R10	No resistance	Non MDR-AB	-	+	-
31	AB045	HA	0	R6	No resistance	Non MDR-AB	+	+	-
32	AB047	HA	0	R11	No resistance	Non MDR-AB	+	+	-
33	AB049	HA	3	R16	AK/CIP/SXT/CTX/CAZ/IPM/MEM/PIP/TE/CRO/FEP/CN	MDR-AB, CR-AB	+	+	-
34	AB050	HA	1	R12	AK/CIP/SXT/CTX/CAZ/CSL/IPM/MEM/PIP/CRO/FEP/CN	MDR-AB, CR-AB	+	-	-
35	AB051	HA	4	R16	AK/CIP/CTX/CAZ/IPM/MEM/PIP/CN	MDR-AB, CR-AB	-	+	-
36	AB053	HA	2	R15	AK/CIP/SXT/CTX/CAZ/CSL/IPM/MEM/PIP/TE/CRO/FEP/CN	MDR-AB,CR-AB, XDR-AB	+	+	*cas1*
37	AB056	HA	0	R6	No resistance	Non MDR-AB	-	+	*cas5, cas6*
38	AB057	HA	2	R15	AK/CIP/SXT/CTX/CAZ/CSL/IPM/MEM/PIP/TE/CRO/FEP/CN	MDR-AB,CR-AB, XDR-AB	+	+	*cas1, cas3*
39	AB058	HA	0	R16	AK/CIP/SXT/CTX/CAZ/IPM/MEM/PIP/CRO/FEP/CN	MDR-AB, CR-AB	-	+	*cas5*
40	AB059	HA	1	R4	AK/CIP/SXT/CTX/CAZ/CSL/IPM/MEM/PIP/TE/CRO/FEP/CN	MDR-AB,CR-AB, XDR-AB	+	+	-
41	AB060	HA	0	R11	No resistance	Non MDR-AB	+	+	-
42	AB061	HA	4	R16	AK/CIP/CTX/CAZ/IPM/MEM/PIP/TE/CRO/FEP/CN	MDR-AB, CR-AB	+	+	-
43	AB063	HA	3	R16	AK/CIP/CTX/CAZ/CSL/IPM/MEM/PIP/TE/CRO/FEP/CN	MDR-AB, CR-AB	+	+	-
44	AB065	HA	0	R16	AK/CIP/SXT/CTX/CAZ/IPM/MEM/PIP/CRO/FEP/CN	MDR-AB, CR-AB	+	+	*cas1, cas3, cas5*
45	AB066	HA	0	R12	CIP/SXT/CTX/CAZ/IPM/MEM/PIP/CRO/FEP/CN	MDR-AB, CR-AB	+	+	-
46	AB067	HA	1	R16	AK/CIP/SXT/CTX/CAZ/IPM/MEM/PIP/CRO/FEP/CN	MDR-AB, CR-AB	-	+	*cas1, cas3, cas5*
47	AB068	HA	1	R16	AK/CIP/SXT/CTX/CAZ/CSL/IPM/MEM/PIP/CRO/FEP/CN	MDR-AB, CR-AB	-	+	*cas1, cas3, cas5*
48	AB069	HA	3	R16	AK/CIP/CTX/CAZ/CSL/IPM/MEM/PIP/TE/CRO/FEP/CN	MDR-AB, CR-AB	+	+	-
49	AB070	HA	1	R16	AK/CIP/SXT/CTX/CAZ/IPM/MEM/PIP/CRO/FEP/CN	MDR-AB, CR-AB	-	+	*cas1, cas3, cas5*
50	AB071	HA	0	R16	AK/CIP/SXT/CTX/CAZ/CSL/IPM/MEM/PIP/CN	MDR-AB, CR-AB	+	+	*cas1, cas3, cas5*
51	AB072	HA	0	R2	No resistance	Non MDR-AB	+	+	-
52	AB074	HA	3	R16	AK/CIP/CTX/CAZ/CSL/IPM/MEM/PIP/TE/CRO/FEP/CN	MDR-AB, CR-AB	+	+	-
53	AB075	HA	2	R16	AK/CIP/CTX/CAZ/CSL/IPM/MEM/PIP/CRO/FEP/CN	MDR-AB, CR-AB	+	+	-
54	AB077	HA	2	R16	AK/CIP/CTX/CAZ/IPM/MEM/PIP/TE/CRO/FEP/CN	MDR-AB, CR-AB	+	+	-
55	AB081	HA	2	R4	AK/CIP/SXT/CTX/CAZ/CSL/IPM/MEM/PIP/TE/CRO/FEP/CN	MDR-AB,CR-AB, XDR-AB	+	+	-
56	AB082	HA	6	R16	AK/CIP/SXT/CTX/CAZ/IPM/MEM/PIP/TE/CRO/FEP/CN	MDR-AB, CR-AB	+	+	-
57	AB084	HA	5	R16	AK/CIP/SXT/CTX/CAZ/IPM/MEM/PIP/TE/CRO/FEP/CN	MDR-AB, CR-AB	+	+	-
58	AB085	HA	2	R16	AK/CIP/SXT/CTX/CAZ/IPM/MEM/PIP/TE/CRO/FEP/CN	MDR-AB, CR-AB	+	+	-
59	AB086	HA	0	R12	CIP/CTX/CAZ/IPM/MEM/PIP/TE/CRO/FEP/CN	MDR-AB, CR-AB	+	+	-
60	AB088	HA	2	R16	AK/CIP/CTX/CAZ/IPM/MEM/PIP/TE/CRO/FEP/CN	MDR-AB, CR-AB	+	+	-
61	AB089	HA	3	R4	AK/CIP/SXT/CTX/CAZ/CSL/IPM/MEM/PIP/TE/CRO/FEP/CN	MDR-AB,CR-AB, XDR-AB	+	+	-
62	AB090	HA	4	R16	AK/CIP/CTX/CAZ/IPM/MEM/PIP/TE/CRO/CN	MDR-AB, CR-AB	+	+	-
63	AB092	HA	1	R27	CIP/CTX/CAZ/CSL/IPM/MEM/PIP/TE/CRO	MDR-AB, CR-AB	+	+	*cas6*
64	AB123	HB	3	R16	CIP/SXT/CTX/CAZ/CSL/IPM/MEM/PIP/TE/CRO/FEP/CN	MDR-AB, CR-AB	+	+	-
65	AB124	HB	0	R12	CIP/CTXCAZ/IPM/MEM/PIP/CRO/FEP	MDR-AB, CR-AB	-	+	-
66	AB125	HB	3	R16	AK/CIP/SXT/CTX/CAZ/IPM/MEM/PIP/TE/CRO/FEP	MDR-AB, CR-AB	+	-	-
67	AB126	HB	3	R16	AK/CIP/SXT/CTX/CAZ/IPM/MEM/PIP/TE/CRO/FEP/CN	MDR-AB, CR-AB	+	+	-
68	AB127	HB	0	R16	CIP/SXT/CTX/CAZ/PIP/TE/CRO/FEP/CN	MDR-AB	+	+	-
69	AB128	HB	5	R16	AK/CIP/SXT/CTX/CAZ/IPM/MEM/PIP/TE/CRO/FEP/CN	MDR-AB, CR-AB	+	+	-
70	AB129	HB	0	R16	AK/CIP/SXT/CTX/CAZ/IPM/MEM/PIP/TE/CRO/FEP/CN	MDR-AB, CR-AB	+	-	-
71	AB130	HB	3	R16	AK/CIP/SXT/CTX/CAZ/IPM/MEM/PIP/TE/CRO/CN	MDR-AB, CR-AB	+	-	-
72	AB131	HB	3	R16	AK/CIP/CTX/CAZ/CSL/IPM/MEM/PIP/TE/CRO/FEP/CN	MDR-AB, CR-AB	+	-	-
73	AB132	HB	3	R16	AK/CIP/SXT/CTX/CAZ/IPM/MEM/PIP/TE/CRO/FEP/CN	MDR-AB, CR-AB	+	+	-
74	AB133	HB	3	R16	AK/CIP/SXT/CTX/CAZ/IPM/MEM/PIP/TE/CRO/FEP/CN	MDR-AB, CR-AB	+	+	-
75	AB134	HB	0	R16	AK/CIP/SXT/CTX/CAZ/IPM/MEM/PIP/CRO/FEP/CN	MDR-AB, CR-AB	-	+	*cas1, cas3, cas5*
76	AB135	HB	3	R4	AK/CIP/SXT/CTX/CAZ/CSL/IPM/MEM/PIP/TE/CRO/FEP/CN	MDR-AB,CR-AB, XDR-AB	+	-	-
77	AB136	HB	0	R16	AK/CIP/SXT/CTX/CAZ/CSL/IPM/MEM/PIP/TE/CRO/FEP/CN	MDR-AB,CR-AB, XDR-AB	-	+	*cas1, cas3, cas5*
78	AB137	HB	1	R11	CIP/SXT/CTX/MEM/CRO	MDR-AB, CR-AB	+	+	-
79	AB138	HB	0	R12	CIP/SXT/CTX/CAZ/CSL/IPM/PIP/TE/CRO/FEP/CN	MDR-AB, CR-AB	+	+	-
80	AB139	HB	9	R11	CIP/CTX/CAZ/CRO/FEP/CN	MDR-AB	+	+	*cas1, cas3, cas5*
81	AB140	HB	4	R4	AK/CIP/SXT/CTX/CAZ/CSL/IPM/MEM/PIP/TE/CRO/FEP/CN	MDR-AB,CR-AB, XDR-AB	+	+	-
82	AB141	HB	0	R12	CIP/SXT/CTX/CAZ/IPM/PIP/TE/CRO/FEP/CN	MDR-AB, CR-AB	+	+	-
83	AB194	HB	0	R12	CIP/CTX/CAZ/IPM/MEM/PIP/TE/CRO/FEP	MDR-AB, CR-AB	+	-	-
84	AB195	HB	0	R16	AK/CIP/SXT/CTX/CAZ/IPM/MEM/PIP/CRO	MDR-AB, CR-AB	-	+	*cas1, cas3, cas5*
85	AB196	HB	0	R12	AK/CIP/SXT/CTX/CAZ/IPM/MEM/PIP/TE/CRO/FEP	MDR-AB, CR-AB	+	-	-
86	AB197	HB	1	R12	CIP/SXT/CTX/CAZ/IPM/MEM/PIP/TE/CRO/FEP	MDR-AB, CR-AB	+	-	-
87	AB198	HB	0	R12	AK/CIP/SXT/CTX/CAZ/IPM/MEM/PIP/TE/CRO/FEP	MDR-AB, CR-AB	+	-	-
88	AB199	HB	3	R16	CIP/SXT/CTX/CAZ/IPM/MEM/PIP/TE/CRO/FEP	MDR-AB, CR-AB	+	+	-
89	AB200	HB	3	R16	CIP/CTX/CAZ/IPM/MEM/PIP/TE/CRO/FEP	MDR-AB, CR-AB	+	+	-
90	AB204	HB	0	R12	CIP/CTX/CAZ/CRO/TE	MDR-AB	-	+	-
91	AB205	HB	0	R4	No resistance	Non MDR-AB	+	-	-
92	AB206	HB	3	R16	AK/CIP/SXT/CTX/CAZ/IPM/MEM/PIP/CRO/FEP/CN	MDR-AB, CR-AB	-	+	*cas1, cas3, cas5*
93	AB208	HB	1	R16	AK/CIP/SXT/CAZ/CSL/IPM/MEM/PIP/TE/CRO/FEP/CN	MDR-AB, CR-AB	+	+	-
94	AB209	HB	3	R16	AK/CIP/SXT/CAZ/CSL/IPM/MEM/PIP/TE/CRO/FEP/CN	MDR-AB, CR-AB	+	+	-
95	AB210	HB	0	R12	AK/CIP/SXT/CAZ/IPM/MEM/PIP/TE/CRO/FEP/CN	MDR-AB, CR-AB	+	-	-
96	AB212	HB	0	R16	AK/CIP/SXT/CAZ/MEM/PIP/CRP/FEP/CN	MDR-AB, CR-AB	-	+	*cas1, cas3, cas5*
97	AB214	HB	0	R12	CIP/SXT/TE	MDR-AB	+	+	-

98	AB215	HB	1	R6	No resistance	Non MDR-AB	+	+	*cas6*
99	AB216	HB	3	R16	AK/CIP/SXT/CAZ/CSL/IPM/MEM/PIP/TE/CRO/FEP/CN	MDR-AB, CR-AB	+	+	-
100	AB217	HB	3	R16	AK/CIP/SXT/CAZ/IPM/MEM/PIP/TE/CRO/FEP/CN	MDR-AB, CR-AB	+	+	-
101	AB219	HB	0	R12	CIP/CAZ/PIP/CRO/FEP	MDR-AB	-	+	-
102	AB220	HB	3	R16	AK/CIP/SXT/CAZ/IPM/MEM/PIP/TE/CRO/CN	MDR-AB, CR-AB	+	-	-
103	AB221	HB	2	R16	AK/CIP/SXT/CAZ/IPM/MEM/PIP/TE/CRO/FEP/CN	MDR-AB, CR-AB	+	+	-
104	AB225	HB	2	R16	AK/CIP/SXT/CAZ/IPM/MEM/PIP/TE/CRO/FEP/CN	MDR-AB, CR-AB	+	-	-
105	AB226	HB	0	R12	CIP/SXT/CTX/CAZ/IPM/PIP/CRO/FEP/CN	MDR-AB, CR-AB	+	+	-
106	AB227	HB	1	R16	CIP/SXT/CTX/CAZ/IPM/MEM/PIP/TE/CRO/FEP/CN	MDR-AB, CR-AB	+	+	-
107	AB228	HB	0	R14	No resistance	Non MDR-AB	-	-	-
108	AB229	HB	3	R4	AK/CIP/SXT/CTX/CAZ/CSL/IPM/MEM/PIP/TE/CRO/FEP/CN	MDR-AB,CR-AB, XDR-AB	+	+	-
109	AB094	HC	0	R13	No resistance	Non MDR-AB	+	+	*cas6*
110	AB095	HC	0	R4	AK/CIP/SXT/CTX/CAZ/CSL/IPM/MEM/PIP/TE/CRO/FEP/CN	MDR-AB,CR-AB, XDR-AB	+	+	-
111	AB097	HC	0	R6	No resistance	Non MDR-AB	+	+	-
112	AB098	HC	0	R12	SXT/CTX/CAZ/CRO/FEP/TE	MDR-AB	-	-	*cas6*
113	AB099	HC	2	R16	AK/CIP/CTX/CAZ/IPM/MEM/PIP/TE/CRO/FEP/CN	MDR-AB, CR-AB	+	+	-
114	AB101	HC	5	R24	AK/CIP/CTX/CAZ/IPM/MEM/PIP/TE/CRO/FEP/CN	MDR-AB, CR-AB	+	+	-
115	AB102	HC	3	R24	IPM/MEM/PIP/TE/CN	MDR-AB, CR-AB	+	+	-
116	AB103	HC	0	R8	IPM/MEM	CR-AB	+	+	*cas2*
117	AB106	HC	0	R6	No resistance	Non MDR-AB	+	-	-
118	AB109	HC	0	R12	CIP/CTX/CAZ/IPM/MEM/PIP/TE/CRO/FEP/CN	MDR-AB, CR-AB	+	+	-
119	AB120	HC	0	R1	CIP/SXT/CTX/CAZ/CSL/IPM/MEM/PIP/CRO/FEP/CN	MDR-AB, CR-AB	+	+	-
120	AB121	HC	3	R24	AK/CIP/CTX/CAZ/IPM/MEM/PIP/TE/CRO/CN	MDR-AB, CR-AB	+	+	-
121	AB143	HC	0	R16	CIP/SXT/CTX/CAZ/IPM/MEM/PIP/TE/CRO/FEP/CN	MDR-AB, CR-AB	-	+	-
122	AB148	HC	0	R1	CIP/SXT/CTX/CAZ/CSL/IPM/MEM/PIP/TE/CRO/FEP/CN	MDR-AB, CR-AB	+	+	-
123	AB159	HC	0	R12	No resistance	Non MDR-AB	+	+	*cas6*
124	AB162	HC	4	R24	AK/CTX/CAZ/CRO/FEP/CIP/CN/IPM/MEM/TE/PIP	MDR-AB, CR-AB	+	+	-
125	AB163	HC	0	R16	CIP/SXT/CTX/CAZ/IPM/MEM/PIP/TE/CRO/FEP	MDR-AB, CR-AB	+	-	-
126	AB166	HC	0	R1	CIP/SXT/CTX/CAZ/IPM/MEM/PIP/CRO/FEP/CN	MDR-AB, CR-AB	+	-	-
127	AB168	HC	0	R1	No resistance	Non MDR-AB	+	+	-
128	AB172	HC	2	R16	AK/CIP/CTX/CAZ/IPM/MEM/PIP/TE/CRO/FEP/CN	MDR-AB, CR-AB	+	+	*cas1*
129	AB175	HC	4	R31	AK/CIP/SXT/CTX/CAZ/IPM/MEM/PIP/TE/CRO/FEP/CN	MDR-AB, CR-AB	+	+	-
130	AB176	HC	5	R24	AK/CIP/CTX/CAZ/IPM/MEM/PIP/TE/CRO/FEP/CN	MDR-AB, CR-AB	+	+	-
131	AB177	HC	5	R24	AK/CIP/CTX/CAZ/IPM/MEM/PIP/TE/CRO/FEP/CN	MDR-AB, CR-AB	+	+	-
132	AB180	HC	0	R4	AK/CIP/SXT/CTX/CAZ/CSL/IPM/MEM/PIP/TE/CRO/FEP/CN	MDR-AB,CR-AB, XDR-AB	+	+	-
133	AB183	HC	3	R4	AK/CIP/SXT/CTX/CAZ/CSL/IPM/MEM/PIP/TE/CRO/FEP/CN	MDR-AB,CR-AB, XDR-AB	+	+	-
134	AB185	HC	1	R12	AK/CIP/SXT/IPM/MEM/PIP/FEP/CN	MDR-AB, CR-AB	-	+	*cas1, cas3, cas5*
135	AB230	HC	5	R24	CIP/CTX/CAZ/IPM/MEM/PIP/TE/CRO	MDR-AB, CR-AB	+	-	-
136	AB231	HC	0	R1	CIP/CTX/CAZ/IPM/MEM/CRO/FEP	MDR-AB, CR-AB	+	+	-
137	AB232	HC	0	R16	CIP/SXT/CTX/CAZ/CSL/IPM/MEM/PIP/TE/CRO/FEP/CN	MDR-AB, CR-AB	+	+	-
138	AB233	HC	2	R24	AK/CIP/SXT/CTX/CAZ/IPM/MEM/PIP/TE/CRO/FEP/CN	MDR-AB, CR-AB	+	+	-
139	AB235	HC	0	R1	No resistance	Non MDR-AB	+	+	*cas5*
140	AB237	HC	3	R16	AK/CIP/SXT/CTX/CAZ/IPM/MEM/PIP/TE/CRO/CN	MDR-AB, CR-AB	+	+	-
141	AB239	HC	0	R16	CIP/CTX/CAZ/IPM/MEM/PIP/CRO/FEP/CN	MDR-AB, CR-AB	+	+	-
142	AB241	HC	5	R4	AK/CIP/SXT/CTX/CAZ/CSL/IPM/MEM/PIP/TE/CRO/FEP/CN	MDR-AB,CR-AB, XDR-AB	+	-	-
143	AB246	HC	0	R4	AK/CIP/SXT/CTX/CAZ/CSL/IPM/MEM/PIP/TE/CRO/FEP/CN	MDR-AB,CR-AB, XDR-AB	+	+	*cas1, cas3, cas5*
144	AB248	HC	0	R12	No resistance	Non MDR-AB	+	+	-
145	AB252	HC	0	R16	AK/CIP/CTX/CAZ/CSL/IPM/MEM/PIP/TE/CRO/FEP/CN	MDR-AB, CR-AB	+	-	-
146	AB253	HC	0	R16	AK/CIP/SXT/CTX/CAZ/IPM/MEM/PIP/TE/CRO/FEP/CN	MDR-AB, CR-AB	+	-	-
147	AB254	HC	0	R21	CIP/SXT/CTX/CAZ/CSL/IPM/MEM/PIP/TE/CRO/FEP/CN	MDR-AB, CR-AB	+	-	-
148	AB255	HC	0	R12	No resistance	Non MDR-AB	+	-	-
149	AB261	HC	0	R2	No resistance	Non MDR-AB	-	+	*cas5*
150	AB265	HC	1	R16	AK/CIP/CTX/CAZ/IPM/MEM/PIP/TE/CRO/FEP/CN	MDR-AB, CR-AB	+	+	-
151	AB266	HC	5	R24	AK/CIP/CTX/CAZ/IPM/MEM/PIP/TE/CRO/CN	MDR-AB, CR-AB	+	+	-
152	AB269	HC	0	R4	AK/CIP/SXT/CTX/CAZ/CSL/IPM/MEM/PIP/TE/CRO/FEP/CN	MDR-AB,CR-AB, XDR-AB	+	-	-
153	AB270	HC	0	R14	No resistance	Non MDR-AB	+	-	-
154	AB275	HC	0	R16	IPM/MEM	CR-AB	+	-	-
155	AB279	HC	5	R28	AK/CIP/CTX/CAZ/IPM/MEM/PIP/TE/CRO/FEP/CN	MDR-AB, CR-AB	+	+	-
156	AB280	HC	3	R21	AK/CIP/SXT/CTX/CAZ/IPM/MEM/PIP/TE/CRO/FEP/CN	MDR-AB, CR-AB	+	+	-
157	AB284	HC	0	R6	No resistance	Non MDR-AB	+	-	-
158	AB287	HC	6	R22	AK/CIP/CTX/CAZ/IPM/MEM/TE/CRO/CN	MDR-AB, CR-AB	+	+	-
159	AB313	HD	0	R1	AK/CIP/TMX/CTX/CAZ/IPM/MEM/PIP/CRO/FEP	MDR-AB, CR-AB	+	+	-
160	AB314	HD	2	R16	CIP/TMX/CTX/CAZ/IPM/MEM/PIP/TE/CRO/FEP/CN	MDR-AB, CR-AB	+	+	-
161	AB316	HD	6	R12	TMX/TE	Non MDR-AB	+	+	-
162	AB318	HD	0	R1	AK/CIP/TMX/CTX/CAZVIPM/MEM/PIP/CRO/FEP	MDR-AB, CR-AB	+	+	-
163	AB319	HD	4	R16	CIP/TMX/CTX/CAZ/IPM/MEM/PIP/TE/CRO/FEP/CN	MDR-AB, CR-AB	+	+	-
164	AB320	HD	5	R4	AK/CIP/TMX/CTX/CAZ/CSL/IPM/MEM/PIP/TE/CRO/FEP/CN	MDR-AB,CR-AB, XDR-AB	+	+	-
165	AB324	HD	0	R4	AK/CIP/TMX/CTX/CAZ/CSL/IPM/MEM/PIP/TE/CRO/FEP/CN	MDR-AB,CR-AB, XDR-AB	-	+	-
166	AB326	HD	0	R16	CIP/TMX/CTX/CAZ/CSL/IPM/MEM/PIP/TE/CRO/FEP/CN	MDR-AB, CR-AB	+	-	-
167	AB327	HD	2	R16	CIP/TMX/CTX/CAZ/CSL/IPM/MEM/PIP/TE/CRO/FEP	MDR-AB, CR-AB	+	+	-
168	AB328	HD	0	R16	CIP/TMX/CTX/CAZ/IPM/MEM/PIP/TE/CRO/FEP	MDR-AB, CR-AB	+	+	-
169	AB329	HD	6	R4	AK/CIP/TMX/CTX/CAZ/CSL/IPM/MEM/PIP/TE/CRO/FEP/CN	MDR-AB,CR-AB, XDR-AB	+	+	-
170	AB334	HD	0	R16	CIP/CTX/CAZ/CSL/IPM/MEM/PIP/TE/CRO/FEP	MDR-AB, CR-AB	+	+	-
171	AB335	HD	0	R4	AK/CIP/TMX/CTX/CAZ/CSL/IPM/MEM/PIP/TE/CRO/FEP/CN	MDR-AB,CR-AB, XDR-AB	+	+	-
172	AB339	HD	0	R12	CIP/TMX/CTX/CAZ/CSL/IPM/MEM/PIP/TE/CRO/FEP	MDR-AB, CR-AB	+	+	-
173	AB340	HD	0	R16	AK/CIP/TMX/CTX/CAZ/CSL/IPM/MEM/PIP/TE/CRO/FEP	MDR-AB, CR-AB	+	+	-
174	AB341	HD	0	R16	CIP/CTX/CAZ/CSL/IPM/MEM/PIP/TE/CRO/FEP	MDR-AB, CR-AB	+	+	-
175	AB348	HD	0	R8	CIP/CTX/CAZ/IPM/MEM/PIP/CRO/FEP	MDR-AB, CR-AB	-	+	-
176	AB349	HD	3	R16	CIP/TMX/CTX/CAZ/IPM/MEM/PIP/TE/CRO/FEP	MDR-AB, CR-AB	+	+	-
177	AB350	HD	2	R4	AK/CIP/TMX/CTX/CAZ/PIP/CT/TGC/TE/CRO/FEP	MDR-AB, CR-AB	+	+	-
178	AB353	HD	4	R16	CIP/TMX/CTX/CAZ/CSL/IPM/MEM/PIP/TE/CRO/FEP	MDR-AB, CR-AB	+	+	-
179	AB354	HD	6	R16	CIP/TMX/CTX/CAZ/IPM/MEM/PIP/TE/CRO/FEP	MDR-AB, CR-AB	+	+	-
180	AB355	HD	3	R16	CIP/TMX/CTX/CAZ/IPM/MEM/PIP/TE/CRO/FEP	MDR-AB, CR-AB	+	+	-
181	AB360	HD	3	R16	CIP/TMX/CTX/CAZ/IPM/MEM/PIP/TE/CRO/FEP	MDR-AB, CR-AB	+	+	-
182	AB361	HD	0	R12	CIP/CTX/CAZ/IPM/MEM/PIP/TE/CRO/FEP	MDR-AB, CR-AB	-	+	-
183	AB367	HD	3	R4	AK/CIP/TMX/CTX/CAZ/CSL/IPM/MEM/PIP/TE/CRO/FEP/CN	MDR-AB,CR-AB, XDR-AB	+	+	-
184	AB368	HD	6	R4	AK/CIP/TMX/CTX/CAZ/CSL/IPM/MEM/PIP/TE/CRO/FEP/CN	MDR-AB,CR-AB, XDR-AB	+	-	-
185	AB369	HD	0	R12	CIP/TMX/CTX/CAZ/PIP/TE/CRO/FEP/CN	MDR-AB	+	+	-
186	AB371	HD	6	R16	AK/CIP/TMX/CTX/CAZ/CSL/IPM/MEM/PIP/TE/CRO/FEP/CN	MDR-AB,CR-AB, XDR-AB	+	-	-
187	AB373	HD	0	R12	CIP/TMX/CTX/CAZ/PIP/TE/CRO/FEP/CN	MDR-AB	+	+	-
188	AB375	HD	0	R5	AK/CIP/TMX/CTX/CAZ/IPM/MEM/PIP/CRO/FEP	MDR-AB, CR-AB	+	+	-
189	AB376	HD	1	R4	CIP/CTX/CAZ/CSL/IPM/MEM/PIP/TE/CRO/FEP	MDR-AB, CR-AB	+	-	-
190	AB377	HD	0	R1	AK/CIP/TMX/CTX/CAZ/IPM/MEM/PIP/CRO/FEP	MDR-AB, CR-AB	+	+	-
191	AB385	HD	6	R4	AK/CIP/TMX/CTX/CAZ/CSL/IPM/MEM/PIP/TE/CRO/FEP/CN	MDR-AB,CR-AB, XDR-AB	+	-	-
192	AB388	HD	0	R16	CIP/TMX/CTX/CAZ/IPM/MEM/PIP/TE/CRO/FEP	MDR-AB, CR-AB	+	-	-
193	AB389	HD	7	R16	CIP/TMX/CTX/CAZ/IPM/MEM/PIP/TE/CRO/FEP	MDR-AB, CR-AB	+	-	-
194	AB394	HD	0	R16	TMX	Non MDR-AB	-	-	-
195	AB396	HD	4	R19	CIP/CTX/CAZ/IPM/MEM/PIP/TE/CRO/FEP	MDR-AB, CR-AB	+	-	-
196	AB403	HD	0	R16	CIP/TMX/CTX/CAZ/CSL/IPM/MEM/PIP/TE/CRO/FEP	MDR-AB, CR-AB	+	+	-
197	AB404	HD	4	R16	CIP/TMX/CTX/CAZ/IPM/MEM/PIP/TE/CRO/FEP	MDR-AB, CR-AB	+	+	-
198	AB405	HD	6	R1	AK/CIP/TMX/CTX/CAZ/CSL/IPM/MEM/PIP/TE/CRO/FEP/CN	MDR-AB,CR-AB, XDR-AB	+	+	-

199	AB410	HD	3	R16	CIP/TMX/CTX/CAZ/CSL/IPM/MEM/PIP/TE/CRO/FEP	MDR-AB, CR-AB	+	+	-
200	AB411	HD	0	R4	CIP/CTX/CAZ/CSL/IPM/MEM/PIP/TE/CRO/FEP	MDR-AB, CR-AB	+	-	-
201	AB301	HD	0	R12	CTX/CAZ/CRO/CIP/CN/IPM/MEM/PIP	MDR-AB, CR-AB	-	+	-
202	AB302	HD	6	R16	AK/CTX/CAZ/CRO/FEP/CIP/CN/IPM/MEM/TMX/TE/ PIP	MDR-AB, CR-AB	+	-	-
203	AB303	HD	0	R16	CTX/CAZ/CRO/CN/ TMX/TE/PIP	MDR-AB	+	+	-
204	AB305	HD	0	R12	CTX/CAZ/CRO/FEP/CIP/CN/TMX/TE	MDR-AB	+	-	-
205	AB310	HD	3	R12	CTX/CAZ/CRO/FEP/CIP/IPM/MEM/TE/PIP	MDR-AB, CR-AB	+	-	-
206	AB412	HF	0	R35	AK/CTX/CAZ/CRO/CIP/CN/IPM/MEM/TMX/PIP	MDR-AB, CR-AB	+	-	-
207	AB415	HF	0	R36	CTX/CAZ/CRO/FEP/CIP/CN/IPM/MEM/TMX/PIP	MDR-AB, CR-AB	+	-	-
208	AB416	HF	0	R37	CTX/CAZ/CRO/FEP/CIP/CN/IPM/MEM/TMX/PIP	MDR-AB, CR-AB	+	-	-
209	AB418	HF	0	R36	CTX/CAZ/CRO/FEP/CIP/CN/IPM/MEM/TMX/PIP	MDR-AB, CR-AB	+	-	-
210	AB419	HF	0	R38	AK/CTX/CAZ/CRO/FEP/CIP/IPM/MEM/TMX/TE/PIP	MDR-AB, CR-AB	+	+	*cas1, cas3, cas5*
211	AB420	HF	0	R38	AK/CTX/CAZ/CRO/FEP/CIP/IPM/MEM/TMX/PIP	MDR-AB, CR-AB	+	+	*cas1, cas3, cas5*
212	AB421	HF	0	R36	AK/CTX/CAZ/CRO/FEP/CIP/IPM/MEM/TMX/TE/PIP	MDR-AB, CR-AB	+	-	-
213	AB422	HF	0	R36	AK/CTX/CAZ/CRO/CIP/CN/IPM/MEM/TMX/PIP	MDR-AB, CR-AB	+	-	-
214	AB423	HF	0	R38	AK/CTX/CAZ/CRO/FEP/CIP/CN/IPM/MEM/TMX/PIP	MDR-AB, CR-AB	+	+	*cas1, cas3, cas5*
215	AB424	HF	0	R35	AK/CIP/TMX/CTX/CAZ/CSL/IPM/MEM/PIP/TE/CRO/FEP/CN	MDR-AB,CR-AB, XDR-AB	+	-	-
216	AB429	HF	0	R36	AK/CTX/CAZ/CRO/FEP/CIP/CN/IPM/MEM/TMX/PIP	MDR-AB, CR-AB	+	-	-
217	AB430	HF	0	R38	AK/CTX/CAZ/CRO/FEP/CIP/IPM/MEM/TMX/TE/PIP	MDR-AB, CR-AB	+	+	*cas1, cas3, cas5*
218	AB431	HF	0	R36	CTX/CAZ/CRO/FEP/CIP/CN/IPM/MEM/TMX/PIP	MDR-AB, CR-AB	+	-	-
219	AB433	HF	0	R37	CTX/CAZ/CRO/FEP/CIP/CN/IPM/MEM/TMX/TE/PIP	MDR-AB, CR-AB	+	+	*cas1, cas3, cas5*
220	AB437	HF	0	R39	No resistance	Non MDR-AB	+	+	*cas1, cas3, cas5*
221	AB439	HF	0	R37	CTX/CAZ/CRO/FEP/CIP/CN/IPM/MEM/TMX/TE/SCF/PIP	MDR-AB, CR-AB	+	+	-
222	AB440	HF	0	R37	CTX/CAZ/CRO/FEP/CIP/CN/IPM/MEM/TMX/TE/SCF/PIP	MDR-AB, CR-AB	+	+	-
223	AB443	HF	0	R37	CTX/CAZ/CRO/CN/FEP/CIP/IPM/MEM/TMX/TE/SCF/PIP	MDR-AB, CR-AB	+	+	-
224	AB446	HF	0	R38	AK/CTX/CAZ/CRO/FEP/CIP/IPM/MEM/PIP	MDR-AB, CR-AB	+	+	*cas1, cas3, cas5*
225	AB448	HF	0	R38	CTX/CAZ/CRO/CIP/IPM/MEM/TMX/PIP	MDR-AB, CR-AB	+	+	*cas1, cas3, cas5*
226	AB449	HF	0	R40	AK/CTX/CAZ/CRO/FEP/CIP/IPM/MEM/TMX/PIP	MDR-AB, CR-AB	+	+	-
227	AB451	HF	0	R35	AK/CTX/CAZ/CRO/FEP/CIP/CN/IPM/MEM/TMX/TE/PIP	MDR-AB, CR-AB	+	+	-
228	AB452	HF	0	R38	AK/CTX/CAZ/CRO/FEP/CIP/IPM/MEM/TMX/PIP	MDR-AB, CR-AB	+	+	*cas1, cas3, cas5*
229	AB455	HF	0	R37	CTX/CAZ/CRO/FEP/CIP/CN/IPM/MEM/TMX/TE/SCF/PIP	MDR-AB, CR-AB	+	+	-
230	AB456	HF	0	R37	CTX/CAZ/CRO/FEP/CIP/CN/IPM/MEM/TMX/TE/SCF/PIP	MDR-AB, CR-AB	+	+	-

[a]AK: amikacin, CTX: cefotaxime, CAZ: ceftazidime, CRO: ceftriaxone, FEP: cefepime, CIP: ciprofloxacin, CN: gentamicin, IMP: imipenem, MEM: meropenem, SXT: trimethoprim/sulfamethoxazole, TE: tetracycline, SCF: cefoperazone/sulbactam and TZP: piperacillin/tazobactam.

Table S2. REP-PCR typing among phage susceptible and resistant *A. baumannii* strains

REP-PCR typing	No. of isolates	
	Phage susceptible (n=107)	Phage resistant (n=123)
R1	1/107 (0.93%)	10/123 (8.13%)
R2	0/107 (0.00%)	2/123 (1.63%)
R3	0/107 (0.00%)	0/123 (0.00%)
R4	18/107 (16.82%)	8/123 (6.50%)
R5	0/107 (0.00%)	1/123 (0.81%)
R6	1/107 (0.93%)	6/123 (4.88%)
R7	0/107 (0.00%)	1/123 (0.81%)
R8	0/107 (0.00%)	3/123 (2.44%)
R9	0/107 (0.00%)	1/123 (0.81%)
R10	0/107 (0.00%)	1/123 (0.81%)
R11	2/107 (1.87%)	2/123 (1.63%)
R12	4/107 (3.74%)	23/123 (18.70%)
R13	0/107 (0.00%)	2/123 (1.63%)
R14	0/107 (0.00%)	2/123 (1.63%)
R15	2/107 (1.87%)	0/123 (0.00%)
R16	59/107 (55.14%)	28/123 (22.76%)
R17	0/107 (0.00%)	0/123 (0.00%)
R18	0/107 (0.00%)	0/123 (0.00%)
R19	1/107 (0.93%)	0/123 (0.00%)
R20	0/107 (0.00%)	0/123 (0.00%)
R21	1/107 (0.93%)	2/123 (1.63%)
R22	1/107 (0.93%)	0/123 (0.00%)
R23	0/107 (0.00%)	0/123 (0.00%)
R24	9/107 (8.41%)	0/123 (0.00%)
R25	0/107 (0.00%)	0/123 (0.00%)
R26	0/107 (0.00%)	0/123 (0.00%)
R27	1/107 (0.93%)	0/123 (0.00%)
R28	1/107 (0.93%)	0/123 (0.00%)
R29	0/107 (0.00%)	0/123 (0.00%)
R30	0/107 (0.00%)	0/123 (0.00%)
R31	1/107 (0.93%)	0/123 (0.00%)
R32	0/107 (0.00%)	0/123 (0.00%)
R33	0/107 (0.00%)	0/123 (0.00%)
R34	5/107 (4.67%)	6/123 (4.88%)
R35	0/107 (0.00%)	3/123 (2.44%)
R36	0/107 (0.00%)	6/123 (4.88%)
R37	0/107 (0.00%)	7/123 (5.69%)
R38	0/107 (0.00%)	7/123 (5.69%)
R39	0/107 (0.00%)	1/123 (0.81%)
R40	0/107 (0.00%)	1/123 (0.81%)

Table S3. List of primers used in this study to detect CRISPR-associated (*cas*) genes

No. multiplex	Target genes	Primer name	Sequence (5'-3')	Templates (Genbank accession no.)	Amplicon size (bp)
Multiplex-1	*cas1*	cas1_506-F	GCTGCGATGCGAATGTTATGT	CP030106.1	506
		cas1_506-R	AGTACCCAAAGTGTCGTCGC		
	cas2	cas2_196-F	CCGCTATGGTGTTTAAGTTACGA	UFJW01000108.1	196
		cas2_196-R	GAAAAACCGCAAGACGGTCA		
	cas3	cas3_850-F	ACTTGATTATGATGCTATGCCCCA	CP030106.1	850
		cas3_850-R	TCGCTCTTGTTCACTGCGTA		
Multiplex-2	*cas5*	cas5_611-F	TGCTTGTGCTTTAGGTGAGCA	JZBV01000044.1	611
		cas5_611-R	CACCGTATGGCTCAATCGCA		
	cas6	cas6_432-F	GGAAACTGCCTGCGCATTAC	CP015121.1	432
		cas6_432-R	ATCCCGCGTTTTCACTCTCC		
	cas9	cas9_247-F	ACTCTCGAAGACAAAGCGCA	CP015145.1	247
		cas9_247-R	TGGTTGCGACCACACAGTTT		
